# Atomic Force Microscopy Demonstrates that Candida glabrata Uses Three Epa Proteins To Mediate Adhesion to Abiotic Surfaces

**DOI:** 10.1128/mSphere.00277-19

**Published:** 2019-05-01

**Authors:** Claire Valotteau, Valeria Prystopiuk, Brendan P. Cormack, Yves F. Dufrêne

**Affiliations:** a Louvain Institute of Biomolecular Science and Technology, Université Catholique de Louvain, Louvain-la-Neuve, Belgium; b Department of Molecular Biology and Genetics, Johns Hopkins University School of Medicine, Baltimore, Maryland, USA; c Walloon Excellence in Life Sciences and Biotechnology (WELBIO), Wavre, Belgium; Carnegie Mellon University

**Keywords:** *Candida glabrata*, adhesion, AFM, *EPA*

## Abstract

The fungal pathogen Candida glabrata can cause both mucosal and disseminated infections. Cell adhesion, a key step in colonization and infection, depends in C. glabrata primarily on the Epa family of cell adhesion proteins. While Epa proteins have been documented to mediate specific adhesion to host glycans, some of them also promote nonspecific adhesion to abiotic surfaces, though this is incompletely understood. Here we address this issue using a combination of genetics and single-cell force measurements. By quantifying the forces driving the attachment of single C. glabrata cells to hydrophobic and hydrophilic substrates, we show that cell adhesion is strongly increased by loss of Sir-mediated silencing. Using a series of mutant strains lacking specific *EPA* genes, we demonstrate unexpectedly that three major Epa proteins, Epa1, Epa6, and Epa7, primarily contribute to both hydrophilic and hydrophobic interactions, suggesting a broad role for the Epa adhesins in mediating specific and nonspecific adherence and implicating Epa genes in biofilm formation on abiotic surfaces.

**IMPORTANCE** Candida glabrata cell wall proteins mediate the attachment of C. glabrata to abiotic surfaces through molecular interactions that are poorly understood. Here, we study the forces engaged in Epa-dependent adhesion using single-cell techniques. Fungal adhesion to hydrophilic and hydrophobic substrates involves mainly three Epa proteins, suggesting a broad role for the Epa adhesins in mediating adherence. These proteins might represent a potential target for the development of innovative antifungal drugs.

## Abstract (precis)

Candida glabrata cell wall proteins mediate the attachment of C. glabrata to abiotic surfaces through molecular interactions that are poorly understood. Here, we study the forces engaged in Epa-dependent adhesion using single-cell techniques. Fungal adhesion to hydrophilic and hydrophobic substrates involves mainly three Epa proteins, suggesting a broad role for the Epa adhesins in mediating adherence. These proteins might represent a potential target for the development of innovative antifungal drugs.

## INTRODUCTION

Candida glabrata is an important fungal pathogen in humans. Normally a commensal, it can cause both superficial mucosal infection and, in immunocompromised patients, serious disseminated infection (1, 2). In Europe and the United States, C. glabrata is responsible for up to 20 to 30% of *Candida* bloodstream infections in hospitalized patients (3–6). C. glabrata virulence is likely related to its ability to adhere specifically to host tissues, and it is known to encode a large repertoire of surface proteins, some of which have been directly implicated in adherence to mammalian cells (7). *Candida* cell surface proteins also promote adhesion and biofilm formation on implanted biomaterials, like prosthetics and catheters (8–14). Studying the mechanisms underlying cell adhesion is important in understanding the development of fungal infections and might inform the development of therapeutic avenues for preventing or treating biofilm infections.

Notably, C. glabrata possesses a family of lectins, encoded by the *EPA* genes, which mediate adherence to host glycans (15–18). Strains encode approximately 20 to 25 *EPA* genes, with the exact number differing between strains (15, 19, 20). Different Epa proteins have different glycan specificities, raising the possibility of environment-specific roles for members of this adhesin family (17, 18, 21). In addition to implicating these Epa adhesins, several studies have implicated other cell wall proteins, including the Awp, Aed, and Pwp proteins, in C. glabrata adherence (7, 22). Importantly, characterized adhesins are all glycosylphosphatidylinositol (GPI)-anchored cell wall proteins (GPI-CWPs). These proteins are covalently anchored through a remnant GPI anchor (present at the C terminus of the protein) to β-glucans in the yeast cell wall (23). The GPI-CWP structure is well adapted to mediate adherence. For example, for the *EPA* genes, the N-terminal lectin domain is followed by a large, low-complexity, glycosylated region that acts to project the N-terminal domain away from the site of cell wall attachment at the C terminus. GPI-CWPs of different lengths might therefore be expected to potentially interact with substrates at different distances from the yeast cell surface (24, 25).

The *EPA* genes, and many additional cell wall protein-encoding genes, are located in the subtelomeric regions of C. glabrata, where they are subject to transcriptional silencing mediated by the Sir complex (16, 26). In the absence of the histone deacetylase Sir2, or in the absence of other components of the subtelomeric silencing machinery, subtelomeric genes become derepressed (16, 26), and importantly, the cells become hyperadherent, due in part to transcriptional derepression of *EPA* gene family members and in particular to derepression of three adhesins encoded by *EPA1*, *EPA6*, and *EPA7* (15).

Candida glabrata infection is often found in the context of fungal biofilms, both on mucosal surfaces and on medical devices, such as urinary or central catheters. How does C. glabrata adhere to abiotic surfaces to initiate biofilm formation? Although our understanding of C. glabrata biofilm formation is incomplete, some studies have indicated a role for subtelomeric cell wall proteins in biofilm formation and adherence to abiotic surfaces. First, mutants that disrupt subtelomeric silencing show increased biofilm production; second, *EPA6* mutants are substantially compromised for biofilm formation (26). In addition to these genetic studies, a previous biophysical analysis documented a strong adhesion of C. glabrata to hydrophobic surfaces and showed that much of that adhesion was mediated also by the Epa6 adhesin (27). These data suggest substantial overlap in the regulation of host cell adherence and the regulation of biofilm formation on abiotic surfaces and implicate subtelomeric genes, including some cell wall proteins and specifically Epa6, in that process. Growth conditions can alter cell surface properties, including expression of cell surface proteins, in some cases through effects on subtelomeric silencing (26, 28, 29). How subtelomeric silencing impacts the expression of cell surface proteins and how that impacts C. glabrata adherence to abiotic surfaces remain to be fully explored.

Here, we study the molecular forces involved in the adhesion of C. glabrata to abiotic surfaces by means of single-cell techniques combined with genetic tools. Using atomic force microscopy (AFM) (30), we quantify the forces between single yeast cells and hydrophobic or hydrophilic substrates. We demonstrate that disruption of subtelomeric silencing has a dramatic effect on surface adhesion, indicating that the key adhesins mediating abiotic adhesion are transcriptionally regulated by the subtelomeric silencing machinery. We also show that three major Epa proteins (Epa1, Epa6, and Epa7) contribute strongly to hydrophilic and hydrophobic adhesion. This was surprising since the Epa proteins are clearly lectins with defined specificities for different glycans. Our result here shows surprisingly that these same proteins mediate nonspecific hydrophobic and hydrophilic interactions, suggesting a broad and underappreciated role for the Epa adhesins in mediating adherence by glycan-independent mechanisms.

## RESULTS

### Disruption of subtelomeric silencing dramatically enhances fungal adhesion.

**FIG 1:**
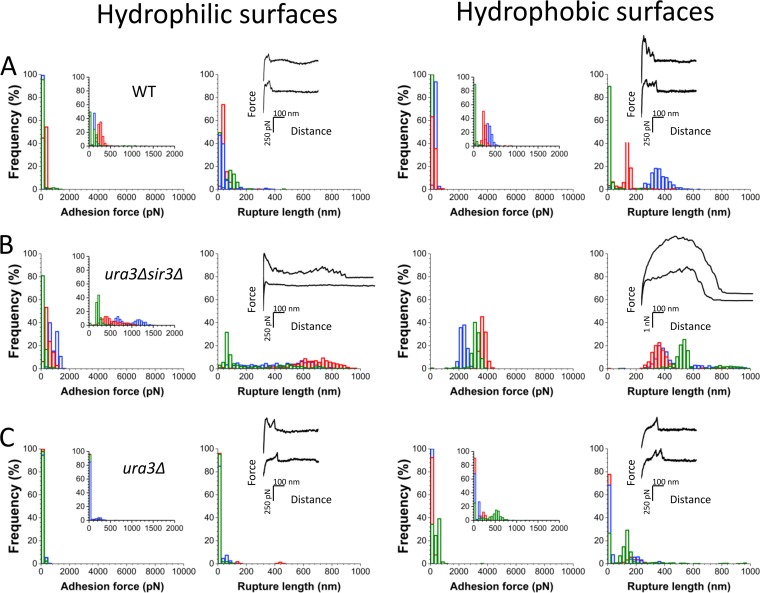
Disruption of subtelomeric silencing dramatically enhances fungal adhesion to abiotic surfaces. (A) AFM-based single-cell force spectroscopy was used to measure the forces between single WT C. glabrata cells and hydrophilic (hydroxyl-terminated) or hydrophobic (methyl-terminated) substrates. Shown here are the adhesion force and rupture length histograms with representative retraction force profiles for three different WT cells interacting with hydrophilic (left) and hydrophobic (right) substrates. (B, C) Force data obtained for the interaction of the *ura3Δ sir3Δ* (B) and *ura3Δ* (C) mutant strains. For results on more cells, see Fig. S1 in the supplemental material.

There is evidence that biofilm formation is increased in mutants that disrupt the silencing machinery (26). We wished therefore to study the adherence of C. glabrata cells to hydrophilic and hydrophobic surfaces, which represents the first step of biofilm formation, and to assess the impact of the loss of subtelomeric silencing on adhesion. We used AFM-based single-cell force spectroscopy (31, 32) to measure the adhesive forces between single C. glabrata cells and hydrophobic (methyl-terminated) or hydrophilic (hydroxyl-terminated) substrates. In Fig. 1A, we show the maximum adhesion forces, rupture lengths, and representative force profiles recorded for three different wild-type (WT) cells (for more cells, see Fig. S1 in the supplemental material). On hydrophilic surfaces (Fig. 1A, left), while a few cells showed poor adhesion, most cells featured moderate adhesion forces (cell 1, 128 ± 46 pN, mean ± standard deviation [SD] from 279 adhesive-force curves; cell 2, 260 ± 66 pN, *n *=* *499; cell 3, 193 ± 190 pN, *n *=* *263). Adherence to hydrophobic surfaces (Fig. 1A, right) also featured moderate adhesion forces (cell 1, 362 ± 64 pN, *n* = 479; cell 2, 248 ± 76 pN, *n* = 443; cell 3, 112 ± 50 pN, *n *=* *14). These data suggest that WT cells, under the growth conditions tested, are only moderately adherent to hydrophilic and hydrophobic surfaces. We note some cell-to-cell variation for adherence to both surfaces (see also Fig. S1), indicating that the cell population is heterogeneous, potentially due to epigenetic regulation of adhesin transcription (see below).

10.1128/mSphere.00277-19.1 FIG S1 Statistical analysis of the interaction forces of the WT, *ura3Δ sir3Δ*, and *ura3Δ* strains. Adhesion forces recorded between hydrophilic (in blue) or hydrophobic (in red) substrates and cells of the WT (*n *=* *1,457 and 3,387 adhesive-force curves, respectively, from 8 cells), *ura3Δ sir3Δ* (*n *=* *997 and 1,299 adhesive-force curves, respectively, from 8 cells), and *ura3* (*n *=* *2,360 and 1,186 adhesive-force curves from 4 cells) strains. Box charts show the mean adhesion (full square), the median, the 25% and 75% quartiles (boxes), and the range of data without outliers (whiskers, 5 to 95 percentiles). Statistical analysis performed by a Student test, with a *P* value level of <0.001, indicated significant differences between all the strains on both types of substrates, except between the WT and *ura3Δ* strains on hydrophobic substrates. Download FIG S1, PDF file, 0.07 MB. Copyright © 2019 Valotteau et al. 2019 Valotteau et al. This content is distributed under the terms of the Creative Commons Attribution 4.0 International license.

To test whether Sir complex-mediated silencing impacts adherence, we analyzed a strain with the *SIR3* gene deleted (this strain also carries a deletion of the *URA3* gene to facilitate genetic manipulation). Notably, adherence in this *ura3Δ sir3Δ* strain was dramatically enhanced for both substrates (Fig. 1B), with the maximum adhesion force increasing up to ∼1,000 pN on hydrophilic surfaces (cell 1, 908 ± 265 pN, *n *=* *508; cell 2, 513 ± 220 pN, *n *=* *507; cell 3, 235 ± 104 pN, *n *=* *475) and to ∼4,000 pN on hydrophobic surfaces (cell 1, 2,306 ± 219 pN, *n *=* *437; cell 2, 3,700 ± 202 pN, *n *=* *508; cell 3, 3,142 ± 430 pN, *n *=* *511). The enhanced adhesion on hydrophobic surfaces shows that the cell surface is engaged in hydrophobic forces, consistent with the ability of C. glabrata to form biofilms on hydrophobic plastic surfaces (8). In addition, for the *sir3Δ* strain, adhesion probability was 97% ± 2% on hydrophilic surfaces and 91% ± 14% on hydrophobic surfaces (versus 58% ± 32% and 91% ± 15%, respectively, in the WT), meaning that adhesive events were observed in almost all curves from all cells. Since the *sir3Δ* mutant strain carries an *ura3* deletion as well, to rule out any role for this mutation, we analyzed *ura3Δ* mutant strains (with intact *SIR3*). Figure 1C shows that the *ura3Δ* mutant behaves like the WT strain, exhibiting moderate and somewhat variable adherence, and thus demonstrating that the dramatic increase in adhesion observed in Fig. 1B results specifically from the disruption of *SIR3* and the loss of SIR complex-mediated transcriptional silencing.

What is the molecular origin of the strong hydrophobic forces? Since the force to unfold and unbind a single β-sheet protein with an AFM probe is ∼250 pN (33), the ∼4,000-pN forces correspond to the simultaneous unbinding and unfolding of multiple cell wall proteins. Consistently with this, we note that longer molecular extensions, up to 1,000 nm, were observed in the hyperadherent *sir3Δ* mutant strain (Fig. 1B), implying that cell detachment involved the unfolding of large cell wall proteins. Similar rupture lengths were observed in Epa6-mediated cell adhesion to hydrophobic substrates (27), leading us to believe that they are associated with Epa proteins. Considering that an amino acid contributes 0.36 nm to the contour length of a fully extended polypeptide chain, the 1,000-nm rupture length suggests that adherence is mediated by proteins with an effective length of ∼3,000 amino acids. Epa proteins, and indeed other subtelomeric GPI-anchored cell wall proteins, have predicted sizes of up to several thousand amino acids (34), consistent with observed adherence being mediated by predicted GPI-CWPs. Alternatively, due to the high surface density of Epa proteins, some adherence might be mediated by protein aggregates, for example, those made up of shed GPI-CWPs anchored to other cell wall proteins. Lastly, force profiles with multiple small peaks were sometimes observed on hydrophilic surfaces (Fig. 1B) but never on hydrophobic ones, consistent with the unfolding of multidomain proteins.

From these results, we conclude that transcriptional derepression of the subtelomeric genes dramatically enhances C. glabrata adhesion to solid surfaces and that these interactions involve hydrophobic binding and the unfolding of multiple large proteins. This suggests strongly that adherence is mediated by one or more subtelomeric genes. The C. glabrata subtelomeres encode large numbers of GPI-CWPs, including many members of the *EPA* adhesin family, and our results raised the possibility that subtelomeric *EPA* genes might mediate the enhanced adhesion to solid surfaces.

### All three of Epa1, Epa6, and Epa7 contribute to hydrophobic and hydrophilic adhesion.

Loss of silencing increases adherence to epithelial cells via increased transcription of *EPA* genes, primarily *EPA1*, *EPA6*, and *EPA7* (15). We sought to assess the roles of these in increased adherence to abiotic hydrophilic and hydrophobic surfaces. For these experiments, all strains lacked *URA3*; in addition, all strains lacked *SIR3* so that they would exhibit the enhanced adherence documented in Fig. 1B. Lastly, different subsets of *EPA* genes were deleted to test the role of those *EPA* genes in adhesion. We initially assessed the adhesion of the *ura3Δ sir3Δ epa1Δ epa6Δ epa7Δ* mutant strain, which is impaired in the expression of all three adhesins. Compared to the *ura3Δ sir3Δ* parent (Fig. 1B), the strain with this triple deletion showed lower adherence to both hydrophilic surfaces (up to ∼500 pN; cell 1, 238 ± 72 pN, *n *=* *492 adhesive curves; cell 2, 187 ± 88 pN, *n *=* *491; cell 3, 148 ± 90 pN, *n *=* *240) and hydrophobic surfaces (up to ∼2,000 pN; cell 1, 1,254 ± 256 pN, *n *=* *485; cell 2, 702 ± 275 pN, *n *=* *503; cell 3, 572 ± 274 pN, *n *=* *512). These observations lead us to believe that hydrophilic and hydrophobic interactions in C. glabrata are in part mediated by the Epa1, Epa6, and/or Epa7 proteins.

**FIG 2:**
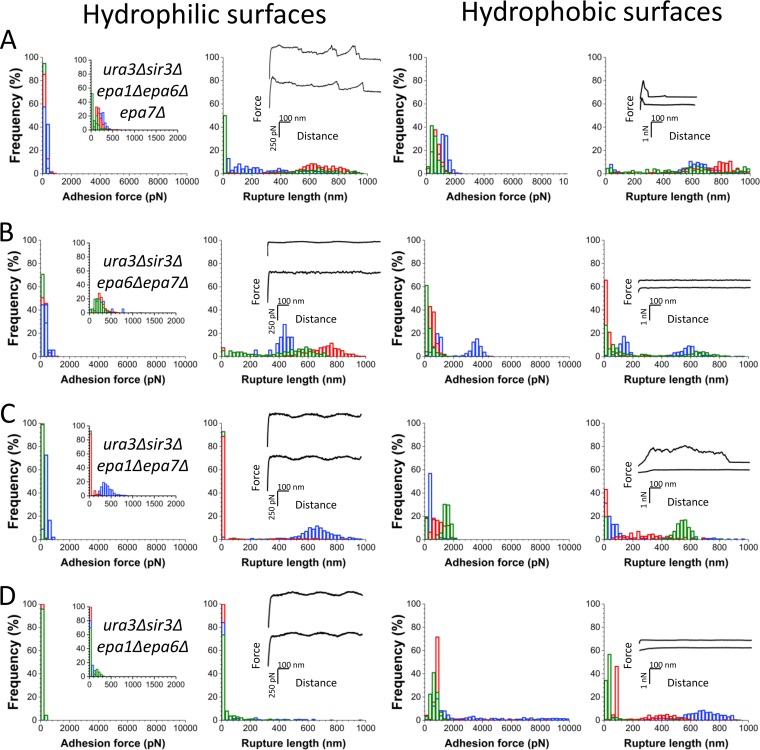
Three adhesins, Epa1, Epa6, and Epa7, contribute to cell adhesion. (A) Adhesion force and rupture length histograms with representative retraction force profiles for three different cells of the *ura3Δ sir3Δ epa1Δ epa6Δ epa7Δ* mutant strain with all three adhesins genes deleted. (B to D) Force data obtained for the interaction of the *ura3Δ sir3Δ epa6Δ epa7Δ* (B), *ura3Δ sir3Δ epa1Δ epa7Δ* (C) and *ura3Δ sir3Δ epa1Δ epa6Δ* (D) mutant strains. For results on more cells, see Fig. S2.

To determine whether any of these three adhesins is sufficient for adherence, we then examined adherence of three double mutant strains with pairs of *EPA1*, *EPA6*, and *EPA7* deleted. Figure 2B to D and Fig. S2 show that, while there were variations from one cell to another, overall, the three mutants showed strongly reduced adherence to both hydrophilic and hydrophobic surfaces. These results show, first, that enhanced adhesion resulting from the loss of subtelomeric silencing depends in part on *EPA1*, *EPA6*, and *EPA7*, implicating Epa1, Epa6, and Epa7 in adhesion to both hydrophobic and hydrophilic surfaces, and, second, that expression of no single one of these genes was sufficient to mediate full adhesion to these abiotic surfaces. Interestingly, for the double-deletion strains, the shapes of the curves on hydrophobic surfaces were clearly different from those of the *ura3*Δ *sir3*Δ parent strain, with multiple rupture peaks, suggestive of the unfolding of single multidomain proteins.

10.1128/mSphere.00277-19.2 FIG S2 Statistical analysis of the interaction forces of the *ura3Δ sir3Δ epa1Δ epa6Δ epa7Δ*, *ura3Δ sir3Δ epa6Δ epa7Δ*, *ura3Δ sir3Δ epa1Δ epa6Δ*, and *ura3Δ sir3Δ epa1Δ epa7Δ* mutant strains. Adhesion forces recorded between hydrophilic (in blue) or hydrophobic (in red) substrates and cells of the *ura3Δ sir3Δ epa1Δ epa6Δ epa7Δ* (*n *=* *2,562 and 3,877 adhesive-force curves from 8 cells), *ura3Δ sir3Δ epa6Δ epa7Δ* (*n *=* *2,669 and 3,057 adhesive-force curves from 8 cells), *ura3Δ sir3Δ epa1Δ epa7Δ* (*n *=* *1,574 and 2,586 adhesive-force curves from 8 cells), and *ura3Δ sir3Δ epa1Δ epa6Δ* (*n *=* *1,236 and 3,826 adhesive-force curves from 8 cells) strains. For comparison, data from the *ura3Δ sir3Δ* strain are also reported here (Fig. 1B and S1). Box charts show the mean adhesion (full square), the median, the 25% and 75% quartiles (boxes), and the range of data without outliers (whiskers, 5 to 95 percentiles). Statistical analysis performed by a Student test, with a *P* value level of <0.001, indicated significant differences between all the strains on both hydrophilic and hydrophobic substrates, except between the *ura3Δ sir3Δ epa1Δ epa7Δ* and *ura3Δ sir3Δ epa1Δ epa6Δ* strains on hydrophilic substrates. Download FIG S2, PDF file, 0.1 MB. Copyright © 2019 Valotteau et al. 2019 Valotteau et al. This content is distributed under the terms of the Creative Commons Attribution 4.0 International license.

### Epa1, Epa6, and Epa7 do not contribute to the moderate adherence of WT strains.

**FIG 3:**
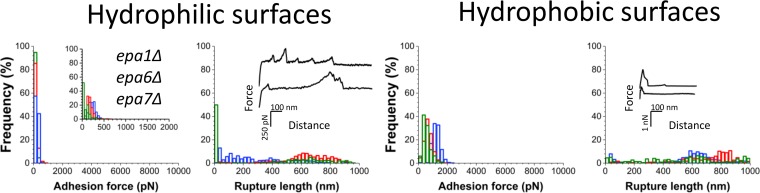
Epa1, Epa6, and Epa7 do not contribute to the moderate adherence of WT strains. Adhesion force and rupture length histograms with representative retraction force profiles for three different cells of the *epa1Δ epa6Δ epa7Δ* mutant strain. For results on more cells, see Fig. S3.

Our data suggest a dramatic adhesion of C. glabrata to abiotic surfaces when silencing of the subtelomeric regions is compromised, mediated in part by Epa1, Epa6, and Epa7. To characterize the impact of these *EPA* genes on adherence in a WT strain background, we compared the WT strain with an *epa1Δ epa6Δ epa7Δ* deletion strain. Figure 3, compared to Fig. 1B, shows that the adhesion forces of the two strains are not substantially different (for more data, see Fig. S3), leading us to conclude that the limited adhesion to abiotic surfaces shown by the WT strain (with silencing intact) is not mediated by the Epa1, Epa6, and Epa7 proteins.

10.1128/mSphere.00277-19.3 FIG S3 Statistical analysis of the interaction forces of the *epa1Δ epa6Δ epa7Δ* mutant strain. Adhesion forces were recorded between hydrophilic (in blue) or hydrophobic (in red) substrates and cells of the *epa1Δ epa6Δ epa7Δ* mutant strain (*n *=* *1,236 and 3,826 adhesive curves, respectively, from 8 cells). For comparison, data from the WT strains are also reported (Fig. 1B and S1). Box charts show the mean adhesion (full square), the median, the 25% and 75% quartiles (boxes), and the range of data without outliers (whiskers, 5 to 95 percentiles). Statistical analysis performed by a Student test, with a *P* value level of <0.001, indicated significant differences between the two strains on both hydrophilic and hydrophobic substrates. Download FIG S3, PDF file, 0.1 MB. Copyright © 2019 Valotteau et al. 2019 Valotteau et al. This content is distributed under the terms of the Creative Commons Attribution 4.0 International license.

## DISCUSSION

The C. glabrata genome encodes approximately 100 GPI-anchored cell wall proteins (34), many of which are hypothesized to contribute to host cell adherence, yeast-yeast interaction, and biofilm formation. One family of GPI-CWPs, the *EPA* genes, encode lectins that mediate adherence to host cells via binding of host glycans. Nonspecific cell-cell interactions, as well as the adherence of C. glabrata to abiotic surfaces, are likely to play important roles in niche colonization, as well as the formation of biofilms either within infected tissue or on medical devices. There is little known about the mechanisms underlying nonspecific cell-cell interaction or biofilm formation. Here, we quantified hydrophilic and hydrophobic interactions between single C. glabrata cells and solid substrates and made two significant findings, (i) adhesion is increased by loss of Sir-mediated silencing and (ii) increased adhesion depends in part on known *EPA* genes and more specifically on the expression of the three major proteins, Epa1, Epa6, and Epa7. That hydrophobic and hydrophilic interactions with abiotic surfaces are dramatically increased by loss of subtelomeric silencing strongly suggests that key genes mediating this nonspecific adherence are carried within the subtelomeric regions normally regulated by the Sir complex. These regions are highly enriched for cell wall protein-encoding genes, making it likely that expression of these CWPs contributes to nonspecific abiotic adherence.

Which of the subtelomeric GPI-CWPs contribute to abiotic adhesion? In *sir3*Δ strains, adherence to mammalian cells is mediated in large part by *EPA* genes and, specifically, *EPA1*, *EPA6*, and *EPA7* (15). Here, we show that the dramatic increase in abiotic surface adhesion found in *sir3*Δ strains also requires these same three genes, implicating them in abiotic adhesion. In addition, we find that expression of any one of these three genes is insufficient to confer the dramatic adhesion of the *sir3*Δ strain, suggesting that adhesion reflects the combined activities of multiple adhesins. That combinatorial action likely includes the actions of subtelomeric cell wall proteins in addition to Epa1, Epa6, and Epa7, since the adhesion of the *ura3Δ sir3Δ epa1Δ epa6Δ epa7Δ* strain to both hydrophobic and hydrophilic surfaces is substantially stronger than the adhesion of the wild-type strain. We do not know the identities of the additional adhesins. The genome encodes 81 adhesin-like GPI-CWPs, of which 50 are encoded in subtelomeric regions. Transcriptional analysis of strains disrupted in subtelomeric silencing show that 40 subtelomeric GPI-CWPs are induced more than 2-fold, with 32 being transcriptionally induced more than 20-fold (Z. Xu and B. P. Cormack, unpublished data). This suggests that in the *sir3Δ* strain, the Epa1, Epa6, and Epa7-independent adherence to hydrophobic and hydrophilic surfaces is likely mediated by additional subtelomeric adhesins, but we are unable to say which specific genes are responsible.

The clear role of Epa1, Epa6, and Epa7 in mediating adhesion to abiotic surfaces was unexpected and surprising and suggests that these proteins, and by implication other Epa adhesins, can mediate interactions via multiple mechanisms. Specifically, our models had focused on Epa-mediated binding to mammalian cells being primarily through a lectin-glycan interaction, with the Epa binding strongly to mammalian glycans. Several lines of evidence show that the lectin activity is primarily responsible for host cell interaction. For example, adherence is reduced by sialylation or by chemical modification of the host glycan (35), and adherence is competed completely by saccharides that correspond to the lectin specificity of particular Epa proteins and not by control saccharides (17, 18, 21). The Epa protein PA14 domain structure has been solved by X-ray crystallography and a clear pocket for glycan docking delineated. The domain structure of the Epa proteins is also consistent with their role as lectins: the N-terminal lectin domain and C-terminal GPI anchor signal are separated by a central domain of variable sequence, which is known to act as a spacer region separating the lectin domain from the cell wall (24).

The sequence and domain structure of the EPA genes is consistent with their role as lectins. Yet, our data here show that these same proteins acting likely synergistically with one another are also responsible for nonspecific robust (with forces in the nanonewton range) hydrophobic and hydrophilic binding. How do these proteins mediate nonspecific binding as well as specific glycan binding? It seems likely that lectin activity *per se* is not required for adherence to abiotic surfaces. How then do Epa1, Epa6, and Epa7 contribute to the striking abiotic adhesion? We speculate that abiotic binding is a function of the large central glycosylated domain of the Epa proteins. This would be consistent with a large body of work with related species implicating different domains of related GPI-CWPs in adherence. For the FLO family lectins of Saccharomyces cerevisiae (25) and the Als adhesins of Candida albicans (36, 37), these central regions have been shown to contribute to adherence indirectly by altering spacing between the N-terminal functional domain and the cell wall attachment site at the C terminus (24) but also directly by less clear mechanisms. The central domains of some Als proteins are important for the protein-protein interactions that underlie Als-mediated amyloid formation (38). Our data are consistent, then, with a model in which particular GPI-CWPs can mediate adhesion by multiple distinct mechanisms. We propose that the Epas are multimodal adhesins and that they can function via specific lectin-glycan interactions mediated by the N-terminal PA14 domain and equally via additional nonspecific hydrophobic or hydrophilic interactions mediated by the large, sometimes extensive, central domains that characterize GPI-CWPs. In this model, the central domains have two potential roles: as spacers to optimize the position of the lectin domain and as direct mediators of robust nonspecific adherence. Specific glycan-mediated interactions and nonspecific hydrophobic and hydrophilic interactions presumably act in concert *in vivo* to confer adherence to a range of different biotic and abiotic surfaces.

In this study, we assessed the adhesion of cells grown to stationary phase in rich media. Under these growth conditions, adhesion was dramatically increased by the loss of subtelomeric silencing, which served to unmask some of the adhesion capacity of the organism. Clearly, environmental conditions which alter the expression of different *EPA* genes will govern the adhesion profile of C. glabrata, and we recognize that a full accounting of the adhesion capacity of C. glabrata must take into account the expression patterns of C. glabrata adhesin genes.

## MATERIALS AND METHODS

### Fungal strains and growth conditions.

C. glabrata strains are described in Table S1 in the supplemental material. C. glabrata strains were grown routinely on YPD (1% yeast extract, 2% peptone, 2% dextrose) agar plates at 37°C. Before all experiments, all strains were incubated in liquid YPD medium overnight at 37°C and grown for at least 16 h into stationary phase. The cells were harvested by centrifugation, washed twice in Hanks’ balanced salt solution (HBSS) supplemented with 5 mM Ca^2+^, and diluted by 100-fold with HBSS.

10.1128/mSphere.00277-19.4 TABLE S1 Fungal strains used in this study. Download Table S1, PDF file, 0.1 MB. Copyright © 2019 Valotteau et al. 2019 Valotteau et al. This content is distributed under the terms of the Creative Commons Attribution 4.0 International license.

### Solid substrates.

Hydrophobic and hydrophilic substrates were prepared by immersing gold-coated substrates in ethanol solutions containing 1 mM 1-dodecanethiol (Sigma-Aldrich; 98%) or 1 mM 11-mercapto-1-undecanol (Sigma-Aldrich; 97%) overnight, by rinsing them with ethanol, and by drying them under N_2_ (27).

### Single-cell force spectroscopy.

For cell probe preparation, wedged cantilevers were prepared using triangular tipless Si_3_N_4_ cantilevers (NP-O10; Bruker) and UV-curable glue (NOA 63; Norland Edmund Optics), according to the methods developed by Alsteens et al. and Stewart et al. (39, 40). These cantilevers were immersed for 1 h in a 200-μg/ml concanavalin A solution, rinsed in HBSS, and then used directly for cell probe preparation. The nominal spring constant of the probe was determined by the thermal noise method. A 50-μl volume of a diluted cell suspension was then deposited into a petri dish containing the hydrophobic and hydrophilic substrates at a distinct location within the petri dish and filled with 3 ml of HBSS auditioned with 5 mM Ca^2+^. The wedged cantilever was brought into contact with an isolated cell and retracted to attach it to the probe; proper attachment of the cell was checked by optical microscopy.

The cell probe was transferred over hydrophilic or hydrophobic substrates without being dewetted. Force measurements were performed at room temperature (20°C) using a Bioscope catalyst AFM (Bruker Corporation, Santa Barbara, CA). A minimum of 100 force distance curves for each cell were recorded on three different spots on a given substrate, with an applied force of 250 pN, a contact time of 100 ms, and constant approach and retract speeds of 1,000 nm s^−1^. Data were analyzed with NanoScape, the data processing software from Bruker. Adhesion and rupture length histograms were generated by considering, for every force curve, the maximum adhesion force and the rupture distance of the last peak.
