# Supplementary figures and images for: Atomic Force Microscopy Demonstrates that Candida glabrata Uses Three Epa Proteins To Mediate Adhesion to Abiotic Surfaces
Source: mSphere. 2019 May 1;4(3):e00277-19. doi: 10.1128/mSphere.00277-19 (PMC6495341; doi:10.1128/mSphere.00277-19)

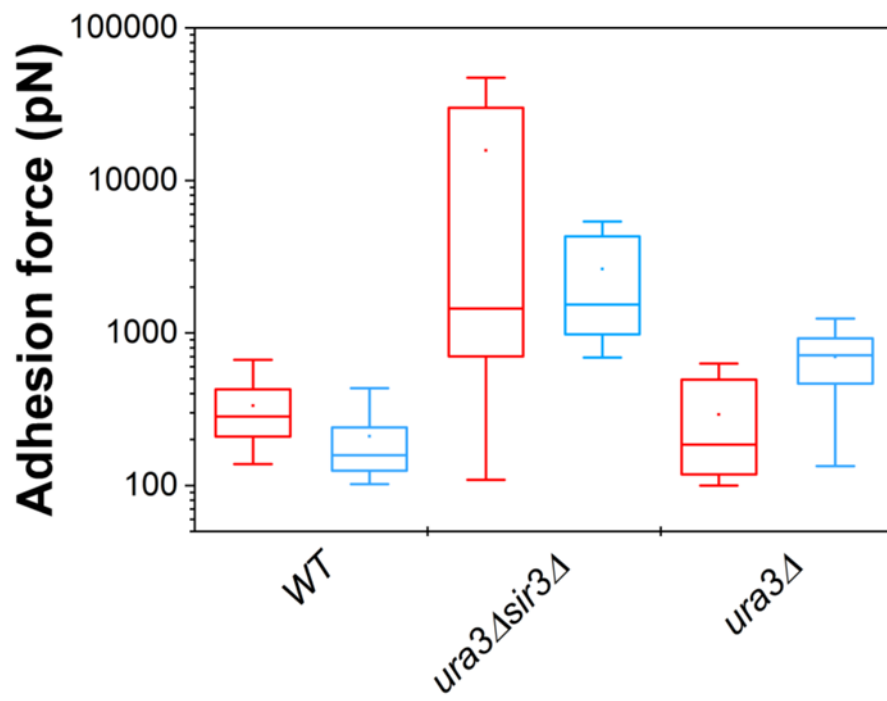

Figure S1

Supplement: FIG S1 [file mSphere.00277-19-sf001.pdf]

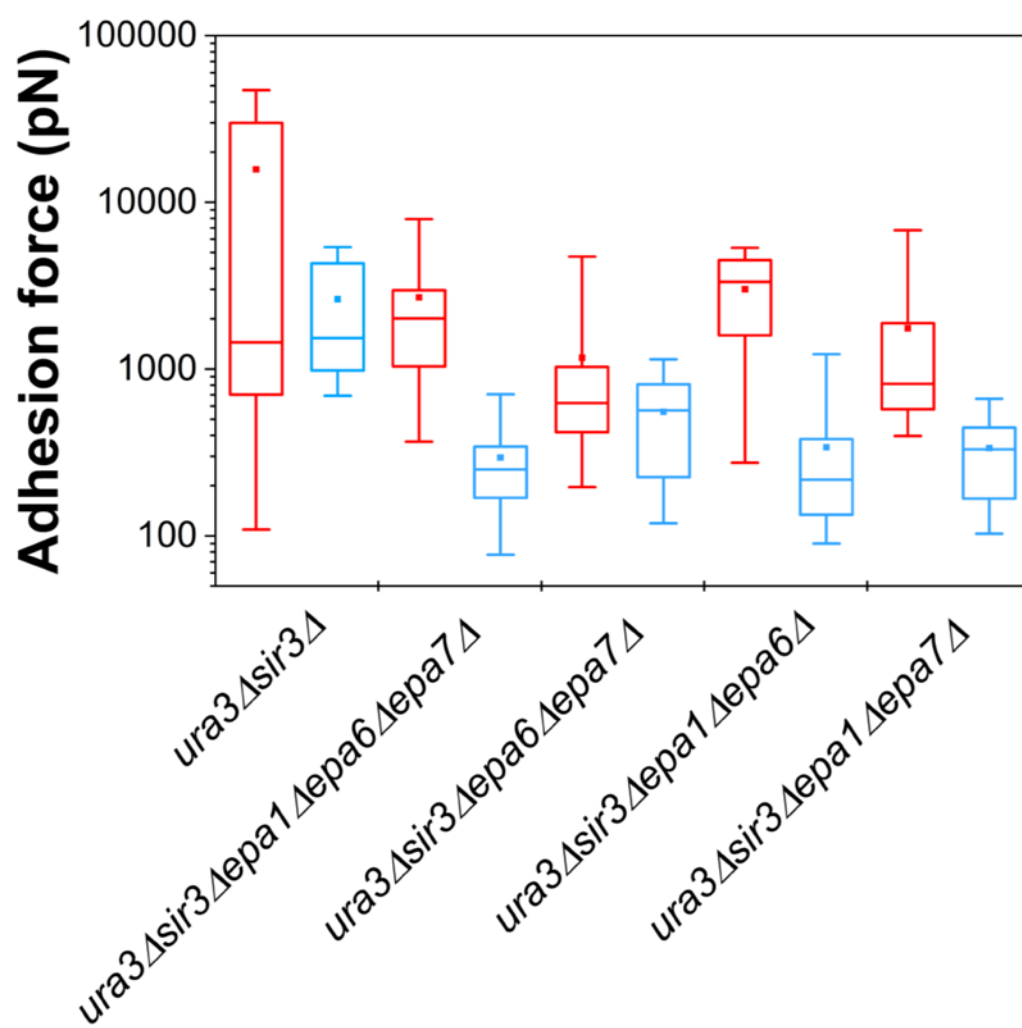

Figure S2

Supplement: FIG S2 [file mSphere.00277-19-sf002.pdf]

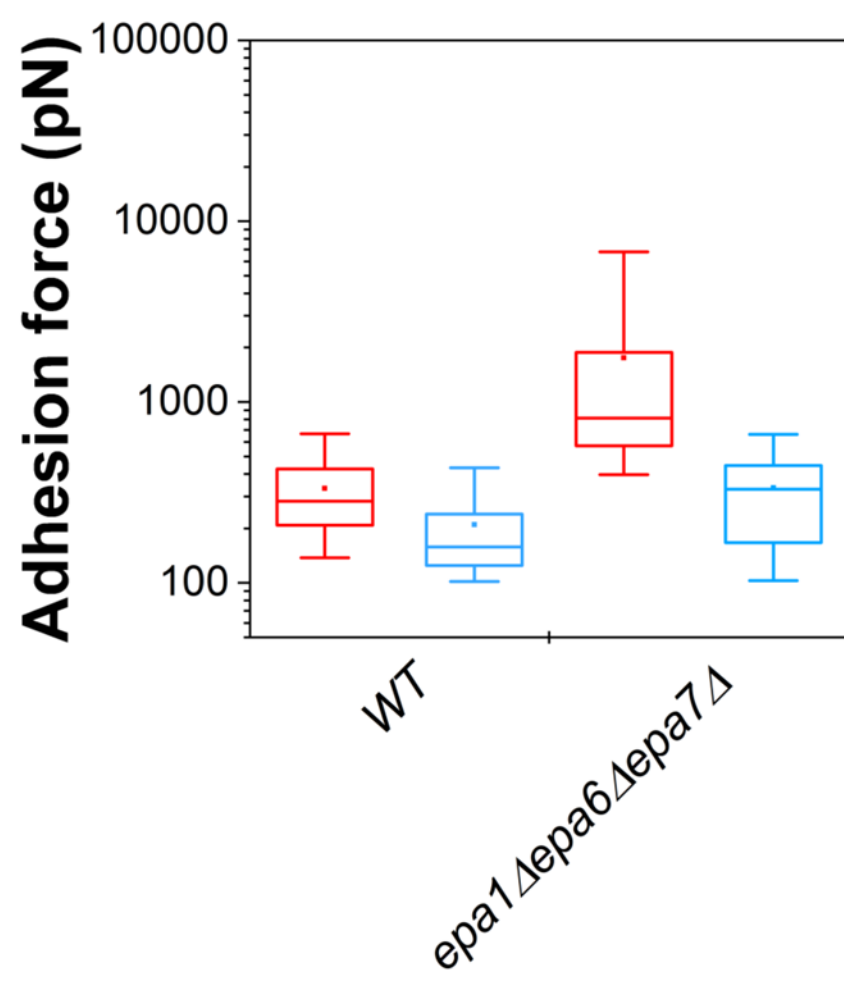

Figure S3

Supplement: FIG S3 [file mSphere.00277-19-sf003.pdf]
