# Supplementary material for: Atomic Force Microscopy Demonstrates that Candida glabrata Uses Three Epa Proteins To Mediate Adhesion to Abiotic Surfaces
Source: mSphere. 2019 May 1;4(3):e00277-19. doi: 10.1128/mSphere.00277-19 (PMC6495341; doi:10.1128/mSphere.00277-19)
Supplement: TABLE S1 [file mSphere.00277-19-st001.pdf]

Table S1. Fungal strains used in this study

Strain	Genotype	
BG2	Clinical isolate	Fidel *et al*, 1996 (1)
BG14	*ura3Δ*::Tn*903* G418^R	Cormack *et al*, 1999 (2)
BG676	*ura3Δ*::Tn*903* G418^R *sir3*Δ	De Las Peñas *et al*, 2003 (3)
BG966	*ura3Δ*::Tn*903* G418^R *sir3Δ* (*epa1*, *epa6*, *epa7)*Δ	Castaño *et al*, 2005 (4)
BG995	*ura3Δ*::Tn*903* G418^R *sir3Δ* (*epa6*, *epa7)*Δ	Castaño *et al*, 2005 (4)
BG1007	*ura3Δ*::Tn*903* G418^R *sir3Δ* (*epa1*, *epa6*)*Δ*	Castaño *et al*, 2005 (4)
BG1012	*ura3Δ*::Tn*903* G418^R *sir3Δ* (*epa1*, *epa7*)*Δ*	Castaño *et al*, 2005 (4)
BG1026	*URA3* (*epa1*, *epa6*, *epa7)*Δ	Castaño *et al*, 2005 (4)
BG1028	*URA3* (*epa1*, *epa7)*Δ	Castaño *et al*, 2005(4)
BG1044	*URA3* (*epa1*, *epa6)*Δ	Castaño *et al*, 2005 (4)
BG1025	*URA3* (*epa6*, *epa7)*Δ	Castaño *et al*, 2005 (4)

1. Fidel PL, Jr., Cutright JL, Tait L, Sobel JD. 1996. A murine model of *Candida glabrata* vaginitis. J Infect Dis 173:425-31.
2. Cormack BP, Falkow S. 1999. Efficient homologous and illegitimate recombination in the opportunistic yeast pathogen *Candida glabrata*. Genetics 151:979-87.
3. De Las Penas A, Pan SJ, Castano I, Alder J, Cregg R, Cormack BP. 2003. Virulence-related surface glycoproteins in the yeast pathogen *Candida glabrata* are encoded in subtelomeric clusters and subject to RAP1- and SIR-dependent transcriptional silencing. Genes Dev 17:2245-58.
4. Castano I, Pan SJ, Zupancic M, Hennequin C, Dujon B, Cormack BP. 2005. Telomere length control and transcriptional regulation of subtelomeric adhesins in *Candida glabrata*. Mol Microbiol 55:1246-58.
